# Assessment of drought tolerance indices in faba bean genotypes under different irrigation regimes

**DOI:** 10.1515/biol-2022-0520

**Published:** 2022-11-14

**Authors:** Manal S. Abdelhaleim, Mehdi Rahimi, Salah A. Okasha

**Affiliations:** Agronomy Department, Faculty of Agriculture, Suez Canal University, 41522, Ismailia, Egypt; Department of Biotechnology, Institute of Science and High Technology and Environmental Sciences, Graduate University of Advanced Technology, Kerman, Iran

**Keywords:** agro-physiological traits, drought, faba bean, tolerance indices

## Abstract

Drought stress has devastating impacts on faba bean production, particularly with the current abrupt climate changes in arid environments. Hence, it is essential to identify drought-tolerant genotypes. The present study aimed at assessing six faba bean genotypes under three irrigation levels during two winter successive growing seasons (2018/2019 and 2019/2020). The applied irrigation levels were well-watered (every 4 days (D1), moderate drought every 8 days (D2), and severe drought 12 days (D3)) regimes. The analysis of variance exhibited highly significant differences among genotypes, irrigation treatments, and their interactions for all studied traits, except the number of pods plant^−1^ in the first season. Yield traits of all assessed genotypes decreased significantly with increasing drought stress. Otherwise, proline content (Pro) increased significantly with increasing drought stress. The genotypes Giza.843, Nubaria.2, and Nubaria.3 recorded the highest values of plant height, number of branches/plant, pods/plant, pods weight/plant, 100 seed weight, seed yield/plant, and seed yield/kg under drought stress. Similarly, the highest Pro was displayed by Giza.843 and Nubaria.3 under drought stress in both seasons. Furthermore, Giza.843, Nubaria.2, and Nubaria.3 genotypes had the highest values for most tolerant indices. Accordingly, these genotypes could be exploited in developing drought-tolerant and high-yielding faba bean genotypes in arid environments through breeding programs.

## 1 Introduction

Faba bean (*Vicia faba* L.) is one of the main legumes grown in Egypt [1]. It is an important protein source for human and animal consumption and plays an important role in crop rotation [2]. Seeds are commonly used as food and feed worldwide and are a major source of protein for over 1 billion people worldwide, but climate change is threatening legume production [3].

However, the total yield of this crop is still not enough to cover local consumption. Globally, the total cultivated area of faba beans was 2.51 million hectares in 2018, producing 4.92 million tons [4]. The total area planted by faba beans in Egypt was small, 40.3 × 103 ha, and yielded 139 × 103 tons. The main producers of faba beans are China, Ethiopia, France, Egypt, and Australia. Faba bean production is not enough to feed the ever-growing world population. Many biological and abiotic factors cause yield loss. In addition, faba bean plants exhibited a large amount of intraspecies variability [5] and molecular and physiological changes occurred [6,7] for stress tolerance. Among cultivated legumes, faba beans are considered vulnerable to water scarcity. Meta-analysis data based on returns from 1980 to 2014 showed that faba bean yields decreased by 40% after a 65% decrease in water availability, with yield losses dependent on cultivar and other environmental conditions [8,9]. Agriculture faced the dual challenges of decreasing crop yields and climate change. Climate change and variability are projected to further reduce agricultural water availability in the future [10,11]. Sustainable crop production requires optimising water use through the development of improved technologies, genotyping, and adaptation strategies. Drought severely affects plant growth, yield, and grain quality and causes morphological, physiological, biochemical, and molecular changes in plants [12,13]. Globally, more than 50% of the average yield of most major crops is lost due to drought stress [14].

Genetic improvement of drought tolerance in faba beans by traditional methods and molecular selection is time and labour-intensive as it is highly dependent on selection and adaptation at multiple sites. Secondary physiological property screening, such as relative water content (RWC), proline, and other physiological properties, can provide generally reliable and repeatable selection information [15,16,17]. Thus, tolerance to stress (TOL) was defined as the difference in yield between stressed (*Y*_S_) and unstressed (*Y*_P_) environments, and mean productivity (MP) was defined as the average yield of *Y*_S_ and *Y*_P_ [18]. The indicators include geometric mean productivity (GMP) [19], mean productivity (MP) [18], harmonic mean (HM) [20], stress sensitivity index (SSI) [21], yield stability index (YSI) [22], yield index (YI) [23], stress tolerance index (STI) [19], relative drought index (RDI) [21], drought response index (DI), and stress sensitivity percentage index (SSPI) [24]. The main objectives of this experiment were to i) determine the difference between faba bean genotypes with different seed yields and their components based on selection indices and ii) determine drought tolerance and susceptibility of genotypes based on physiological and biochemical parameters.

## 2 Materials and methods

### 2.1 Plant material and the irrigation treatments

To achieve the goal of this study, we collected six faba bean genotypes from different geographic regions (Table 1). The six genotypes were evaluated at three levels of water stress (4 days (D1) as controls, after 8 days (D2) as moderate, and 12 days (D3) as severe water stress) in field trials during two winter seasons, 2018/2019 and 2019/2020. The experimental design was a split–plot with three replications. Irrigation treatments were located in the main plots, whereas the cultivars were randomised in subplots. Field trials were performed on loamy clay soil (25.4% sand, 34.2% silt, and 33.6% clay). Some physical and chemical properties of soil composition are provided in Table 2. In both experimental years, genotype seeds were sown in rows in the first week of November at 50 and 20 cm intervals, respectively, and sown on the hill.

**Table 1:** Name and pedigree for the faba bean parental varieties under investigation

Genotypes	Origin	Pedigree
Misr.1	FCRI1	(123A/45/76XG.3) × (62/1570/66 × G.2) × (Romi × Habashi)
Giza.3	EGYPT	G.1*NA 29
Sakha.1	EGYPT	Giza 716 × 620/283/85
Nubaria.3	EGYPT	Selection in Ahnasiaz
Giza.843	FCRI1	Cross 461 × cross 561
Nubaria.2	EGYPT	ILB1550 × Radiation2095/76

FCRI = Field Crops Research Institute, Agricultural Research Center, Giza, Egypt.

**Table 2:** Physical and chemical properties of soil compositions

Type of soil	CaCo_3_ (g/kg)	Ec (ds/m)	pH	Cations (meq/L)				Anions (meq/L)		
				Ca^+^	Mg^+^	Na^+^	K^+^	Cl^−^	{\text{SO}}_{4}^{2-}]	HCO_3_^−^
Loamy clay	0.52	1.4	7.93	3.4	2.7	5.3	0.60	7.5	3.3	0.8

## 3 Data recorded

At maturity (140 days), ten plants from each plot were selected from the middle row and the following traits were measured after harvest: plant height (PH), number of branches per plant (NB), number of pods per plant (PP), pods weight/plant (PW), 100 seed weight (SW), seeds yield/plant (SYP), and seed yield (equal 4,200 m^2^) (plots were harvested on 15 April in both growing seasons).

**Physiological characters (after 60 days**): Some biochemical components, chlorophyll content as soil plant analysis development (SPAD) values, RWC, and proline content (Pro) were determined.

**Chlorophyll SPAD value:** SPAD (SPAD502 chlorophyll Meter, Minolta Co., Ltd., Japan) is a portable, self-calibrating, easy-to-use, non-destructive device that can be used to measure the amount of chlorophyll present in the leaves of plants during their flowering period [25].

**RWC:** Fresh leaf samples (100 mg) were soaked in 10 ml of distilled water until saturated and left overnight. After removing water from the leaf surface without pressure, the leaf was dried at 70°C for 72 h and weighed to obtain a saturated weight. The dry weight was obtained. From these data, RWC was calculated according to Galmés et al. [26] using the following equation:

(1) \text{RWC\%}=(\text{Fresh}\hspace{.25em}\text{weight}\hspace{.25em}-\hspace{.25em}\text{Dry}\hspace{.25em}\text{weight})/(\text{Turgid}\hspace{.25em}\text{weight}\hspace{.25em}-\hspace{.25em}\text{Dry}\hspace{.25em}\text{weight})\times 100.]

### 3.1 Proline concentration

The concentration of proline in the capsule filling step of each site was determined by Yooyongwech et al. [27]. First, a fresh leaf sample was homogenised in 3% sulfosalicylic acid, then ninhydrin and 2 ml of glacial acetic acid were added, and the sample was heated to 100°C. The mixture was then extracted with toluene, and free toluene was quantified spectrophotometrically at 520 nm using l-proline as standard. The proline concentration was determined using a calibration curve and expressed as µg/g fresh leaf weight.

### 3.2 Statistical analysis

Data from two seasons were provided for analysis of variance (two-way ANOVA in a spilt–plot design) with three replicates and after checking for compatibility of errors. Treatments were compared using the least significant difference (L.S.D) at the 5% probability level using AGRI-STAT software. Tolerance indices are determined as presented in Table 3.

**Table 3:** List of drought tolerance indices used for evaluation of faba bean genotypes to drought conditions

Drought tolerance indices	Equation	Outcome	Equation no.
SSI	\text{SSI}=\frac{1-\left(\frac{{Y}_{S}}{{Y}_{P}}\right)}{1-\left(\frac{{\bar{Y}}_{S}}{{\bar{Y}}_{P}}\right)}]	The genotypes with SSI < 1 are more resistant to drought stress conditions	(2)
TOL	\text{TOL}={Y}_{P}-{Y}_{S}]	The genotypes with low values of this index are more stable in two different conditions	(3)
MP	\text{MP}=\frac{{Y}_{S}+{Y}_{P}}{2}]	The genotypes with high value of this index will be more desirable	(4)
GMP	\text{GMP}=\sqrt{({Y}_{S})({Y}_{P})}]	The genotypes with high value of this index will be more desirable	(5)
STI	\text{STI}=\frac{({Y}_{S})({Y}_{P})}{{({\bar{Y}}_{P})}^{2}}]	The genotypes with high STI values will be tolerant to drought stress	(6)
YI	\text{YI}=\frac{{Y}_{S}}{{\bar{Y}}_{S}}]	The genotypes with high value of this index will be suitable for drought stress condition	(7)
YSI	\text{YSI}=\frac{{Y}_{S}}{{Y}_{P}}]	The genotypes with high YSI values can be regarded as stable genotypes under stress and non-stress conditions	(8)
HM	\text{HM}=\frac{2({Y}_{P}\cdot {Y}_{S})}{{Y}_{P}+{Y}_{S}}]	The genotypes with high HM value will be more desirable	(9)
Sensitivity drought index (SDI)	\text{SDI}=\frac{{Y}_{\text{P}}-{Y}_{\text{S}}}{{Y}_{\text{P}}}]		(10)
DI	\text{DI}={Y}_{\text{S}}\times \left[\frac{({Y}_{\text{S}}/{Y}_{\text{P}})}{{\bar{Y}}_{\text{S}}}\right]]		(11)
RDI	\text{RDI}=\frac{({Y}_{\text{S}}/{Y}_{\text{P}})}{({\bar{Y}}_{\text{S}}/{\bar{Y}}_{\text{P}})}]		(12)
SSPI	\text{SSPI}=\left[\frac{{Y}_{p}-{Y}_{s}}{2\times {\bar{Y}}_{p}}\right]\times 100]		(13)

*Y*_S_ and *Y*_P_ are stress and optimal yield of a given genotype, respectively. {\bar{Y}}_{p}] and {\bar{Y}}_{S}] are average yield of all genotypes under optimal and stress conditions, respectively.

## 4 Results

ANOVA for the two seasons revealed very significant differences between genotypes, drought stress, and interactions for all traits except for the pods of plant in the first season (Table 4). In this study, the performance of various genotypes was evaluated by PH, NB, PP, (PW), 100, SYP, seed yield/ard.feddan (SYF), SPAD values, RWC, and Pro (Tables 5–7). The overall improvement in seed growth and yield characteristics was found to be significantly maximal in the D1 treatment, which deteriorated with increasing drought stress. In terms of PH, for irrigated wells, the highest yielding genotypes Nubaria.2, Nubaria.3, and Giza.843 showed a significant decrease when grown under stress conditions compared to the other genotypes (Table 5).

**Table 4:** Mean squares of all the studied traits recorded for faba bean genotypes under non-saline and saline conditions in 2018/19 and 2019/20 seasons

Traits	d.f	2	5		2	10	34
	Seasons	Blocks	Genotypes (*G*)	Error a	Drought (*D*)	*G* × *D*	Error b
PH	S1	9.23	434.34**	9.58	673.49**	24.55**	6.65
	S2	0.75	211.75**	2.72	167.8.82**	28.75**	1.05
NB	S1	25.01	25.71	2.98	65.76	32.63	2.32
	S2	22.15	3.27**	2.05	10.65**	0.15**	2.02
PP	S1	0.66	7.79**	1.16	70.99**	2.65**	0.76
	S2	0.05	48.02**	0.16	76.11**	0.75**	0.13
PW	S1	1.75	2037.31**	0.99	1020.87**	40.74**	0.97
	S2	1.85	2164.57**	1.07	1085.11**	43.25**	1.03
100 SW	S1	2.28	891.40**	1.13	1181.18**	20.22**	0.59
	S2	0.84	378.04**	1.58	1442.23**	21.58**	1.04
SYP	S1	2.16	841.86**	3.16	1132.04**	7.55**	1.36
	S2	1.72	826.66**	3.11	1048.38**	8.52**	1.22
SYF	S1	0.29	113.70**	0.23	390.49**	68.07**	0.18
	S2	0.07	5.45**	0.18	47.42**	1.76**	0.11
Chlorophyll SPAD	S1	25.87	173.64*	54.18	185.78*	138.70*	42.34
	S2	0.12	175.06**	0.21	305.12**	53.46**	0.11
RWC	S1	6.05	100.91**	17.08	3170.97**	114.03**	15.52
	S2	6.85	104.13**	17.69	3010.29**	113.85**	15.98
Pro	S1	2.42	234.15**	1.67	7649.12**	35.96**	1.51
	S2	2.48	227.80**	1.61	7790.01**	35.80**	1.39

S1; season 2018/2019 and S2; 2019/2020.

*; **; means significant differences at *P* value < 0.05 and < 0.01.

**Table 5:** Mean performance of PH, NB, and PP under different irrigation regimes in both seasons

	Irrigation interval				Irrigation interval			
Genotypes	D1	D2	D3	Mean	D1	D2	D3	Mean
**Plan height (cm)**								
Misr.1	89.33	77.33	75.33	80.67	86.67	83.00	76.33	82.00
Giza.3	87.00	73.67	70.67	77.11	91.33	88.67	79.33	86.44
Sakha.1	84.67	72.33	73.33	76.78	82.67	81.00	78.33	80.67
Nubaria.3	98.25	84.50	75.72	86.15	99.24	91.17	83.32	91.24
Giza.843	95.34	79.84	71.36	82.18	98.98	93.08	81.12	91.06
Nubaria.2	101.29	88.59	76.72	88.87	107.27	96.53	94.48	99.42
Mean	92.65	79.38	73.85		94.36	88.91	82.15	
RD%		14.323	20.284			5.780	12.937	
L.S.D 0.05	*G* = 2.62	*I* = 1.85	*G* × *I* = 3.16		*G* = 1.19	*I* = 0.84	*G* × *I* = 1.84	
**NB**								
Misr.1	5.51	4.69	4.22	4.81	4.53	3.53	2.33	3.46
Giza.3	4.53	3.80	3.50	3.95	5.93	4.03	2.60	4.19
Sakha.1	5.82	5.15	4.52	5.17	4.40	4.17	2.50	3.69
Nubaria.3	6.17	5.30	4.43	5.30	5.47	5.08	3.84	4.80
Giza.843	6.57	5.60	4.60	5.59	4.79	4.50	5.53	4.94
Nubaria.2	6.40	5.73	4.43	5.52	5.61	5.67	3.66	4.98
Mean	5.83	5.05	4.29		5.12	4.50	3.41	
RD%		13.487	26.536			36.861	52.118	
L.S.D 0.05	*G* = 5.13	*I* = 3.63	*G* × *I* = 3.63		*G* = 4.39	*I* = 2.12	*G* × *I* = 3.27	
**PP**								
Misr.1	16.37	14.17	12.70	14.41	16.33	14.43	11.17	13.98
Giza.3	20.40	18.17	15.33	17.97	18.17	15.43	11.83	15.14
Sakha.1	18.37	16.33	13.30	16.00	17.40	15.00	12.23	14.88
Nubaria.3	14.04	12.11	10.82	12.33	14.26	13.01	11.86	13.04
Giza.843	14.35	11.38	10.22	11.98	14.26	13.32	11.52	13.04
Nubaria.2	14.94	13.36	11.41	13.24	15.93	14.33	13.92	14.73
Mean	16.41	14.25	12.30		16.06	14.25	12.09	
RD%		13.145	25.061			11.240	24.715	
L.S.D 0.05	*G* = 0.90	*I* = 0.63	*G* × *I* = 0.63		*G* = 0.86	*I* = 0.56	*G* × *I* = 1.37	

**Table 6:** Mean performance of PW, 100 SW, SYP, and SYF under different irrigation regimes in both seasons

	Season 1				Season 2			
Genotypes	Irrigation interval				Irrigation interval			
	3 days	5 days	7 days	Mean	3 days	5 days	7 days	Mean
**PW (g)**								
Misr.1	60.27	51.20	43.57	51.68	34.00	30.33	24.00	29.44
Giza.3	32.67	27.36	24.32	28.12	38.00	33.33	27.67	33.00
Sakha.1	38.23	34.08	29.40	33.90	35.67	32.33	25.33	31.11
Nubaria.3	45.71	39.31	35.14	40.05	38.39	42.36	38.70	39.82
Giza.843	78.60	66.13	53.93	66.22	33.97	32.04	27.82	31.28
Nubaria.2	73.70	63.67	49.63	62.33	41.17	37.08	36.07	38.11
Mean	54.86	46.96	39.33		36.87	34.58	29.93	
RD%		14.405	28.306			6.200	18.808	
L.S.D 0.05	*G* = 0.95	*I* = 0.67	*G* × *I* = 0.73		*G* = 0.98	*I* = 0.69	*G* × *I* = 1.09	
**100 SW (g)**								
Misr.1	76.33	72.00	65.33	71.22	76.67	74.00	61.00	70.56
Giza.3	84.00	76.67	69.00	76.56	86.33	75.33	65.00	75.56
Sakha.1	86.33	81.00	71.00	79.44	86.00	77.00	66.67	76.56
Nubaria.3	96.63	83.45	74.67	84.92	95.39	90.35	82.41	89.38
Giza.843	83.05	69.73	62.34	71.71	86.42	81.41	70.92	79.58
Nubaria.2	98.26	86.55	74.91	86.57	104.89	94.23	92.76	97.30
Mean	87.44	78.23	69.54		89.28	82.05	73.13	
RD%		10.526	20.47			8.098	18.098	
L.S.D 0.05	*G* = 0.83	*I* = 0.58	*G* × *I* = 0.69		*G* = 1.04	*I* = 0.74	*G* × *I* = 1.52	
**SYP (g)**								
Misr.1	24.92	20.78	13.48	19.73	25.42	22.78	12.74	20.31
Giza.3	31.15	27.80	16.65	25.20	32.81	26.45	18.21	25.82
Sakha.1	26.08	22.20	12.83	20.37	28.20	23.18	14.15	21.84
Nubaria.3	47.36	42.76	31.66	40.59	45.80	41.08	33.20	40.03
Giza.843	49.35	44.03	30.55	41.31	50.32	44.90	32.18	42.47
Nubaria.2	40.89	34.89	22.00	32.59	42.12	35.18	24.15	33.82
Mean	36.63	32.08	21.20		37.45	32.26	22.44	
RD%		12.419	42.130			13.843	40.077	
L.S.D 0.05	*G* = 1.31	*I* = 0.93	*G* × *I* = 1.54		*G* = 1.28	*I* = 0.91	*G* × *I* = 1.35	
**Seed yield**								
Misr.1	12.24	10.46	7.74	10.14	12.13	10.20	7.53	9.95
Giza.3	12.60	11.03	8.01	10.55	12.43	10.80	7.89	10.37
Sakha.1	12.31	10.70	7.81	10.28	12.25	10.59	7.62	10.15
Nubaria.3	12.72	11.54	10.64	11.63	31.95	11.84	10.83	18.21
Giza.843	12.49	11.55	10.23	11.42	26.98	11.79	10.25	16.34
Nubaria.2	12.84	11.76	11.39	12.00	12.94	11.63	11.42	12.00
Mean	12.53	11.17	9.30		18.11	11.14	9.26	
RD%		10.831	25.754			38.488	48.884	
L.S.D 0.05	*G* = 0.45	*I* = 0.31	*G* × *I* = 0.72		*G* = 0.54	*I* = 3.12	*G* × *I* = 2.61	

**Table 7:** Mean performance of chlorophyll SPAD values, RWC, Pro, SYP, and seed yield under different irrigation regimes in both seasons

	Season 1				Season 2			
Genotypes	Irrigation interval			Mean	Irrigation interval			Mean
	3 days	5 days	7 days		3 days	5 days	7 days	
**Chlorophyll SPAD values**								
Misr.1	55.57	47.58	42.58	48.58	56.06	51.63	47.20	51.63
Giza.3	62.53	56.21	56.18	58.30	63.75	59.08	59.08	60.64
Sakha.1	62.92	59.46	59.31	60.56	64.25	62.44	62.52	63.07
Nubaria.3	51.88	61.04	54.62	55.85	52.68	57.43	64.13	58.08
Giza.843	60.54	50.81	45.46	52.27	63.03	60.08	51.84	58.32
Nubaria.2	59.51	52.46	45.41	52.46	63.44	57.20	55.64	58.76
Mean	58.82	54.59	50.59		60.54	57.98	56.74	
RD%		7.190	13.993			4.225	6.278	
L.S.D 0.05	*G* = 6.83	*I* = 4.82	*G* × *I* = 5.19		*G* = 5.19	*I* = 4.31	*G* × *I* = 4.69	
**RWC**								
Misr.1	78.44	69.23	64.77	70.82	75.04	66.20	62.37	67.87
Giza.3	82.42	67.83	45.77	65.34	78.62	64.50	43.00	62.04
Sakha.1	84.23	56.79	53.53	64.85	80.67	53.56	51.00	61.74
Nubaria.3	74.77	71.10	45.54	63.80	70.93	66.86	41.67	59.82
Giza.843	72.11	66.17	53.38	63.89	68.72	62.97	50.60	60.76
Nubaria.2	85.89	71.27	55.61	70.92	81.77	67.57	52.00	67.11
Mean	79.64	67.07	53.10		75.96	63.61	50.11	
RD%		15.791	33.329			16.256	34.034	
L.S.D 0.05	*G* = 3.91	*I* = 2.76	*G* × *I* = 2.78		*G* = 3.97	I = 2.80	*G* × *I* = 2.71	
**Pro**								
Misr.1	46.97	66.44	81.78	65.06	45.33	65.00	80.67	63.7
Giza.3	52.32	74.88	94.18	73.79	50.50	73.33	93.00	72.3
Sakha.1	49.76	66.97	97.52	71.42	48.33	65.67	96.33	70.1
Nubaria.3	55.14	73.50	98.89	75.84	53.40	72.00	97.67	74.4
Giza.843	58.26	79.77	93.39	77.14	56.67	78.33	92.00	75.7
Nubaria.2	44.26	65.00	88.15	65.80	42.83	63.67	87.00	64.5
Mean	51.12	71.09	92.32		49.51	69.67	91.11	
RI%		39.1	80.6			40.7	84.0	
L.S.D 0.05	*G* = 1.22	*I* = 0.86	*G* × *I* = 1.48		*G* = 1.19	*I* = 0.84	*G* × *I* = 2.26	

Mean values for the PH were 92.7, 94.4 cm in D1, 79.4, 88.9 cm in D2, and 73.9, 82.2 cm in D3 with a decreasing percentage of about 14.3, 5.8% and 20.3, 12.9% than D1 overall genotypes in both seasons, respectively. The tallest genotypes across three irrigation treatments were Nubaria.2 (88.9, 99.4 cm), Nubaria.3 (86.15, 91.24 cm), and Giza.843 (82.18, 91.06 cm), while the shortest was Sakha.1 where it recorded 76.78, 80.67 (cm) in both seasons. For the NB, all genotypes had the highest number of branches under D1 and decreased gradually with increasing drought stress (Table 5). Mean values for PH were 5.83, 7.12 in D1, 5.05, 4.50 in D2 and 4.29, 3.41 in D3 with a decreasing percentage of about 13.48, 36.86% and 26.53, 52.12% than D1 overall genotypes in both seasons, respectively. The genotypes Giza.843, Nubaria.2, and Nubaria.3 had the highest number of branches under all drought treatments with mean values of 5.59, 5.52, and 5.30 in season 2017/18 and 4.94, 4.98, and 4.80 in season 2018/19, respectively. All genotypes produced the highest NB in D1 and decreased significantly with increasing drought stress. The maximum PP was recorded by Giza.3 followed by Sakha.1 with values of 17.97, 15.14 and 16.00, 17.88 in the first and second seasons, respectively. Concerning the PW, all genotypes had higher values under well irrigation than in other conditions; however, the PW was decreased by about 14.40, 6.20 and 28.30, 18.81% in D1 and D2 at first and second seasons, respectively. The heaviest genotype for the PW was Giza.843 (66.22 g) followed by Nubaria.2 (62.33 g) in the first season and Nubaria.3 (39.82 g) and Nubaria.2 in the second season. On the other hand, Giza.3 and Sakha.1 in first season and Misr.1 and Sakha.1 in the second season were the light genotypes than others (Table 6). Concerning 100 seeds plant^−1^, all genotypes produced the highest values under well-watered conditions (D1), but under severe stress, all genotypes decreased by about 20.47 and 18.08 in D2 and D3 in the first and second seasons, respectively. Nubaria.2 shared Nubaria.3 in the first rank with mean 86.57, 97.30 and 84.92, 89.38 in both seasons, respectively (Table 6). Concerning SYP, all genotypes recorded high values under well irrigation and decreased significantly in other conditions; however, SYP was decreased about 12.419, 42.130% and 13.843, 40.077% under D2 and D3 at first and second seasons, respectively. The heaviest genotype as a grand mean was Giza.843 (41.31, 42.47 g) followed by Nubaria.3 (42.47, 40.03 g) in both seasons. On the other hand, in both conditions, Misr.1 was the light genotype and the same direction was obtained in the SYP for the seed yield, where the Giza.843 and Nubaria.3 cultivars were the best genotypes in this trait, and the least of them was the Misr.1 under all stress conditions in the two seasons (Table 6).

All genotypes recorded high values for SPAD values under D1 and decreased gradually with increasing drought stress, except Misr.1 under D2 (Table 7). In both seasons, the mean chlorophyll SPAD values were 58, 82, and 60.54 in D1, 54.59, 57.98 in D2, and 50.59, 56.74 in D3, with a decreasing percentage of about 7.19, 4.22%, and 13.99, 6.27% than in D1 overall genotypes. The genotype Sakha.1 had the highest values of chlorophyll SPAD values in overall drought treatments, with mean values of 60.56 and 63.07 in the first and second seasons as a grand mean, respectively, while the lowest one was Misr.1 in both seasons. In Table 7, leaf RWC differed between cultivars and was significantly reduced under water-deficit stress. Genotypes decreased significantly with increasing drought stress. However, 79.64 and 75.96 in D1, 67.07, 63.61 in D2, and 53.10, 50.11 in D3 with a decreasing percentage of about 15.79, 33.32% and 16.25, 34.03% in both seasons compared to D1 overall genotypes. The maximum value of RWC was observed in Nubaria.2 followed by Misr.1. On the other hand, Giza.843 had the lowest genotype than others in both conditions. Pro for all genotypes increased significantly with increasing drought stress. Moreover, the increasing percentage was 39.06, 80.06, and 40.72, 84.02 in D2 and D3 compared with D1 in both seasons. The highest Pro was displayed by Giza.843 (77.14, 75.7) and Nubaria.3 (75.84, 74.40) under all environmental conditions in both seasons (Table 7).

### 4.1 Tolerance indices of six faba bean cultivars grown under moderate and severe drought stress

The results shown in Table 8 in moderate (D2) and severe (D3) drought-stressed faba bean cultivars Misr1 and Sakha.1 exhibited the lowest TOL values (tolerance index), whereas the highest values of these indices were Nubaria.2 and Giza.843 in both conditions. Nubaria.3 and Giza.843 showed the lowest SSI values and they were less than the unit (<1.0) in D2 and D3. The highest MP, GMP, STI, YI, YSI, and HM indices values were recorded by Nubaria.3 and Giza.843, while the cultivars Misr.1 and Sakha.1 had the lowest values for these indices. The highest SDI indices were recorded by Sakha.1, whereas the lowest values were recorded in Nubaria.3, followed by Giza.843. According to the DI index, all genotypes had values less than unity except Nubaria.3 and Giza.843. Based on RDI, two genotypes, Nubaria.3 and Giza.843, had values more than one in both conditions, and Misr.1, only the cultivar gave equally one in D2. Faba bean cultivars Nubaria.2 and Giza.843 exhibited the highest SSPI values, whereas the least values were recorded by Misr.1, followed by Sakha.1.

**Table 8:** Tolerance indices of six faba bean cultivars grown under D2 (regular) and D3 (bold) of water requirement as an average of SYP across two seasons

	Misr.1	Giza.3	Sakha.1	Nubaria.3	Giza.843	Nubaria.2
Yp‡	*25.17*	*31.98*	*27.14*	*46.58*	*49.84*	*41.51*
Ys‡	21.78	27.13	22.69	41.92	44.47	35.04
	13.11	17.43	13.49	32.43	31.37	23.08
TOL‡	3.39	4.86	4.45	4.66	5.37	6.47
	12.06	14.55	13.65	14.15	18.47	18.43
SSI‡	1.03	1.16	1.25	0.76	0.82	1.19
	1.17	1.11	1.22	0.74	0.90	1.08
MP‡	23.48	29.55	24.92	44.25	47.15	38.27
	19.14	24.71	20.32	39.51	40.60	32.29
GMP‡	23.41	29.45	24.82	44.19	47.07	38.13
	18.17	23.61	19.13	38.87	39.54	30.95
STI‡	0.40	0.63	0.45	1.42	1.62	1.06
	0.24	0.18	0.12	0.49	0.51	0.31
YI‡	0.68	0.84	0.71	1.30	1.38	1.09
	0.60	0.80	0.62	1.49	1.44	1.06
YSI‡	0.87	0.85	0.84	0.90	0.89	0.84
	0.52	0.55	0.50	0.70	0.63	0.56
HAM‡	23.35	29.35	24.72	44.13	47.00	38.00
	17.24	22.56	18.02	38.24	38.50	29.66
SDI‡	0.13	0.15	0.16	0.10	0.11	0.16
	0.48	0.45	0.50	0.30	0.37	0.44
DI‡	0.59	0.72	0.59	1.17	1.23	0.92
	0.31	0.44	0.31	1.03	0.90	0.59
RDI	1.00	0.98	0.96	1.04	1.03	0.97
	0.88	0.93	0.84	1.18	1.07	0.94
SSPI‡	4.58	6.55	6.01	6.29	7.25	8.73
	16.28	19.64	18.43	19.10	24.94	24.88

‡Yp = Seed yield/plant (g) under control; Ys = Seed yield/plant under drought stress (D2% or D3%); TOL = tolerance to stress; SSI = Stress sensitivity index; MP = mean productivity; GMP = Geometric mean productivity; STI = Stress tolerance index; YI = Yield index; YSI = Yield stability index; HAM = Harmonic mean; SDI = Sensitivity drought index; DI = Drought resistance index; SSPI = Stress sensitivity percentage index.

## 5 Discussion

Drought is one of the major factors of biological stress that affects almost all plant functions [28]. The effects of water scarcity on physiological and biochemical processes, growth, and yield processes for various crops have been thoroughly discussed and analysed [16,17,28]. This study showed that drought stress significantly reduced pH, grain yield, and their component characteristics (Tables 5–7). Among crops, faba beans are considered more susceptible to drought than other grain legumes [15]. The plant’s developmental stage and the magnitude of water deficit determine the yield loss of the faba bean. The most susceptible stages for developmental inhibition have been variously described as flowering [29], early podding [30], and pod setting [31], but all of these studies generally agree that the early reproductive phase is the most sensitive stage [19,32]. Moderate drought stress had a negative effect on the PP but had no effect on the seed size or seed number per pod [29,33].

Drought stress negatively affected all faba bean genotypes in our study. Growth parameters of the cultivar decreased when stressed due to water deprivation compared to controls, which may be related to tumour loss and decreased RWC. Drought issues can reduce cell division and elongation, reducing PH and leaf area. In this study, water stress showed a very significant difference in all properties studied (Tables 5 and 6). Also, water stress is highly dependent on the number of pods. On the other hand, the interaction between cultivars and watering interval showed significant differences for all yields and yield components studied. A decrease in PH may be associated with insufficient water absorption and a decrease in photosynthetic efficiency [34]. They concluded that higher cultivars had a greater ability to support seed sowing in stem reserves under drought conditions due to greater storage space. Therefore, it can be said that there is no limit to the selection of the highest plant genotype under drought conditions.

Lack of water in the rooting environment can cause reduced reproduction of the reproductive system. This decline may be due to the cumulative effect of various factors leading to the reduced number of flowers and faulty development of pollen grains and ovules, resulting in improper fertilization and denature of embryos [35,36]. Failure to sow seeds in plants and obtain small, light seeds in water-scarce conditions leads to inconsistencies in the reproduction growth of these plants. Drought is known to be associated with plant growth because it reduces the availability of water to plants. It influences photosynthesis and nitrogen metabolism, as well as the activity of several enzymes involved in seed growth and development [37].Given the fact that yield is essential for growth throughout the season, characteristics that affect a plant’s ability to survive or during periods of water scarcity may be relatively important for drought tolerance [38]. To maximise seed yield in drought conditions, the ability to efficiently transfer nutrients from stems and leaves to growing seeds is desirable [35,37]. A major factor in growth and yield decline is primarily related to the limited supply of metabolic energy to maintain normal growth processes. Drought increases the amount of work required to respond to osmotic and ionic stress for normal cell maintenance, leaving less energy for growth needs as a result. Our results are consistent with [39], which shows that faba bean growth is profoundly affected by stress levels due to water scarcity. Golabadi et al. [40] studied the effect of water stress on two cultivars of faba bean with different growth habits and found that PH and number of pods significantly responded to water stress.

RWC is a robust and simply assessable selection criterion that can describe the plant’s water status to the metabolism irrespective of plant parts and species. It can be expressed as the water content of tissues in normal conditions compared to hydrated conditions [41]. During water-deficit conditions, the RWC plays an important role by preserving water (stomatal features, leaf area reduction, and leaf dropping) or maximising water absorption (root plasticity). Link et al. [42] found the RWC superior to water potential for assessing plant water status. Under nonstress conditions, Khazaei et al. [43] identified the RWC as one of the most important traits that distinguished wet- and dry-adapted faba bean accessions. Cell wall rigidity relates to both the solute concentrations and the cell wall rigidity [44]. The RWC can efficiently identify drought-tolerant genotypes based on their plant water status in faba beans [5,19,45], common beans [46], and chickpeas [47,48]. Therefore, it can be said that genotypes that can sustain a higher RWC in a water-deficit environment would be suitable for use in breeding faba beans for drought adaptation.

Under drought stress, leaf RWCs play an important role in plant stress resistance by inducing osmotic regulation through the accumulation of osmoprotectants [5]. Maintaining a high moisture state of plants during stress is essential to maintaining sufficient moisture. Stomata closure, reduced leaf area, senescence of old leaves, and so on increased water uptake (e.g. increased root growth) [35]. In the present experiment, Table 7 shows that leaf RWC, Pro accumulation, and total chlorophyll content (SPAD values) of all genotypes were significantly affected by water stress. As the drought-induced stress level increased, the RWC and total chlorophyll contents in the leaves decreased inversely. Differences in RWCs in all genotypes may be related to their ability to absorb water from the soil. Therefore, we conclude that the genotypes “Giza843, Nubaria.2 and Nubaria.3” may have a better ability to withstand drought stress.

Proline is found to be the most prevalent amino acid found in plant tissues under stress conditions (drought, cold, and salinity). Singh et al. [36] demonstrated a correlation between drought and an increased free proline accumulation in drought-tolerant barley cultivars compared to a more drought-susceptible genotype. Accumulation of proline in plants reduces the toxic effect of ions on enzyme activity and also reduces the formation of free radicals formed as a result of stress [49]. Proline is also associated with regenerative resistance, serving as a source of respiration energy in stressed plants [12]. Free proline in leaves increased due to drought stress (Table 7). However, significant genetic variation in the genotypes tested under osmotic stress and drought conditions was observed, possibly due to disruption of water flow from the xylem to the surrounding renal cells [14,15,40].

Venekamp et al. [50] found that just 1 day of water deficit at the seedling stage induced proline accumulation. An increased proline concentration in faba beans was observed with the increase of stress intensity, and the variation in proline concentrations at the genotypic level was reported to be low under optimal conditions [17,51,52]. However, an exogenous proline application can decrease stomatal opening under drought, and this has a positive impact on drought tolerance mechanisms [53]. Proline accumulation provides an indication of the plant’s physiological status, i.e. whether it is stressed or not, but not a descriptive drought tolerance indicator in faba beans.

A decrease in chlorophyll content (Table 7) under drought stress may be associated with accelerated leaf aging, which, as observed in this study (Table 7), shows the rate of light assimilation [37], and thus grain yield may have decreased. These indicators made it possible to identify superior genotypes under conditions of drought stress. DSI, YSI, GMP, and MP parameters were related to yield under stress conditions, suggesting that these constructs are suitable for testing drought tolerance and high-yield processing under stress conditions [36,37]. Adaptation factors, rather than drought tolerance, may account for potential yield differences [32]. The effect of drought on crop productivity depends on the severity of the drought and the stage of plant growth in which it occurs [54].

Finally, no single trait and approach are adequate to improve yields under drought conditions, the most complex environmental factor for faba bean productivity. A combination of screening methods suitable for specific environments and expansion of the scale of breeding will be required to allow expansion of the production area and yield of the faba bean, a crop that is an increasingly important source of plant-based protein in drought-prone production regions.

## 6 Conclusion

Faba bean is more sensitive to drought than other field crops, and reduced yields are positively related to the amount of water available. However, this study highlights the need to prioritise the selection and development of genotypes for drought-resistant fab bean. SYP was high in all genotypes under well irrigation and decreased significantly with increasing water irrigation interval, but it was decreased under D2 and D3 in the first and second seasons, respectively. Also, the highest Pro was displayed by Giza.843 and Nubaria.3 under all environmental conditions in the two seasons. Giza.843 and Nubaria.3 cultivars were the best genotypes for physiological and agronomic characters in all conditions, and they gave the highest values for most tolerant indices, making them suitable for drought stress conditions. Therefore, we highly recommend that future faba bean breeding programmes use the tolerance indices parameters to account for multiple traits in multienvironmental trials.

## References

[1] Anil KS, Naresh C, ra M, Anitha P. An assessment of faba bean (Vicia faba L.) current status and future prospect. Afr J Agric Res. 2013;8(50):6634–41.

[2] Askar A. Food science: Faba beans (Vicia faba L.) and their role in the human diet. Food Nutr Bull. 1986;8(3):1–11.

[3] Mansour E, Mahgoub HA, Mahgoub SA, El-Sobky ES, Abdul-Hamid MI, Kamara MM, et al. Enhancement of drought tolerance in diverse Vicia faba cultivars by inoculation with plant growth-promoting rhizobacteria under newly reclaimed soil conditions. Sci Rep. 2021;11(1):1–20. 10.1038/s41598-021-02847-2 PMC8683512 34921154

[4] FAOSTAT. Statistical databases. Food and Agriculture Organization of the United Nations, https://wwwfaoorg/faostat/en/#data/QCL, (Accessed april, 2021); 2020.

[5] Siddiqui MH, Al-Khaishany MY, Al-Qutami MA, Al-Whaibi MH, Grover A, Ali HM, et al. Response of different genotypes of faba bean plant to drought stress. Int J Mol Sci. 2015;16(5):10214–27. 10.3390/ijms160510214 PMC4463642 25950766

[6] Cortés AJ, Monserrate FA, Ramírez-Villegas J, Madriñán S, Blair MW. Drought tolerance in wild plant populations: the case of common beans (Phaseolus vulgaris L.). PLoS One. 2013;8(5):e62898. 10.1371/journal.pone.0062898 PMC3643911 23658783

[7] Kabbadj A, Makoudi B, Mouradi M, Pauly N, Frendo P, Ghoulam C. Physiological and biochemical responses involved in water deficit tolerance of nitrogen-fixing Vicia faba. PLoS one. 2017;12(12):e0190284. 10.1371/journal.pone.0190284 PMC5744999 29281721

[8] Daryanto S, Wang L, Jacinthe P-A. Global synthesis of drought effects on cereal, legume, tuber and root crops production: A review. Agric Water Manag. 2017;179:18–33.

[9] Muktadir MA, Adhikari KN, Merchant A, Belachew KY, Vandenberg A, Stoddard FL, et al. Physiological and biochemical basis of faba bean breeding for drought adaptation–A review. Agronomy. 2020;10(9):1345.

[10] Calzadilla A, Rehdanz K, Betts R, Falloon P, Wiltshire A, Tol RS. Climate change impacts on global agriculture. Clim Change. 2013;120(1):357–74.

[11] Farooq M, Hussain M, Siddique KH. Drought stress in wheat during flowering and grain-filling periods. Crit Rev Plant Sci. 2014;33(4):331–49.

[12] Abid G, Hessini K, Aouida M, Aroua I, Baudoin J-P, Muhovski Y, et al. Agro-physiological and biochemical responses of faba bean (Vicia faba L. var.‘minor’) genotypes to water deficit stress. Biotechnol Agron Soc Envir. 2017;21(2):146–59.

[13] Qabil N, Helal A, El-Khalek A, Rasha Y. Evaluation of some new and old faba bean cultivars (Vicia faba L.) for earliness, yield, yield attributes and quality characters. Zagazig J Agric Res. 2018;45(3):821–33.

[14] Zlatev Z, Lidon FC. An overview on drought induced changes in plant growth, water relationsand photosynthesis. Emir J Food Agric. 2012;24(1):57–72.

[15] Bista DR, Heckathorn SA, Jayawardena DM, Mishra S, Boldt JK. Effects of drought on nutrient uptake and the levels of nutrient-uptake proteins in roots of drought-sensitive and-tolerant grasses. Plants. 2018;7(2):28. 10.3390/plants7020028 PMC6027393 29601475

[16] Ghaffari H, Tadayon MR, Nadeem M, Razmjoo J, Cheema M. Foliage applications of jasmonic acid modulate the antioxidant defense under water deficit growth in sugar beet. Span J Agric Res. 2019;17:e0805.

[17] Migdadi H, El-Harty E, Salamh A, Khan M. Yield and proline content of faba bean genotypes under water stress treatments. J Anim Plant Sci. 2016;26(6):1772–9.

[18] Rosielle A, Hamblin J. Theoretical aspects of selection for yield in stress and non‐stress environment 1. Crop Sci. 1981;21(6):943–6.

[19] Fernandez GC, editor. Effective selection criteria for assessing plant stress tolerance. Proceeding of the International Symposium on Adaptation of Vegetables and other Food Crops in Temperature and Water Stress, Aug 13–16, Shanhua, Taiwan; 1992.

[20] Jafari A, Paknejad F, Jami Al-Ahmadi M. Evaluation of selection indices for drought tolerance of corn (Zea mays L.) hybrids. Int J Plant Prod. 2009;3(4):33–8.

[21] Fischer R, Maurer R. Drought resistance in spring wheat cultivars. I. Grain yield responses. Aust J Agric Res. 1978;29(5):897–912.

[22] Bouslama M, Schapaugh W. Stress tolerance in soybean. Part 1: evaluation of three screening techniques forheat and drought tolerance. Crop Sci. 1984;24:933–7.

[23] Gavuzzi P, Rizza F, Palumbo M, Campanile R, Ricciardi G, Borghi B. Evaluation of field and laboratory predictors of drought and heat tolerance in winter cereals. Can J Plant Sci. 1997;77(4):523–31.

[24] Mousavi S, YAZDI SB, Naghavi M, Zali A, Dashti H, Pourshahbazi A. Introduction of new indices to identify relative drought tolerance and resistance in wheat genotypes. Desert. 2008;12:165–78.

[25] Minolta K. Chlorophyll meter SPAD-502 instruction manual. Minolta Co., Ltd., Radiometric Instruments Operations Osaka, Japan; 1989. p. 22.

[26] Galmés J, Flexas J, Savé R, Medrano H. Water relations and stomatal characteristics of Mediterranean plants with different growth forms and leaf habits: responses to water stress and recovery. Plant Soil. 2007;290(1):139–55.

[27] Yooyongwech S, Cha-um S, Supaibulwatana K. Proline related genes expression and physiological changes in indica rice response to water-deficit stress. Plant Omics. 2012;5(6):597–603.

[28] Shakeel AA, Xiao-yu X, Long-chang W, Muhammad FS, Chen M, Wang L. Morphological, physiological and biochemical responses of plants to drought stress. Afr J Agric Res. 2011;6(9):2026–32.

[29] Zarafshar M, Akbarinia M, Askari H, Hosseini SM, Rahaie M, Struve D, et al. Morphological, physiological and biochemical responses to soil water deficit in seedlings of three populations of wild pear (Pyrus boisseriana). Biotechnol Agron Soc Envir. 2014;18:353–66.

[30] Blum A. Drought resistance–is it really a complex trait? Funct Plant Biol. 2011;38(10):753–7. 10.1071/FP11101 32480932

[31] Singh B. Mutations in crop improvement. In: Singh B, editor. Plant breeding: principles & methods. Ludhiana: Kalyani publishers; 2016.

[32] Fang X, Turner NC, Yan G, Li F, Siddique KH. Flower numbers, pod production, pollen viability, and pistil function are reduced and flower and pod abortion increased in chickpea (Cicer arietinum L.) under terminal drought. J Exp Bot. 2010;61(2):335–45. 10.1093/jxb/erp307 PMC2803204 19854801

[33] Munns R. Physiological processes limiting plant growth in saline soils: some dogmas and hypotheses. Plant Cell Env. 1993;16(1):15–24.

[34] Emam Y, Shekoofa A, Salehi F, Jalali A. Water stress effects on two common bean cultivars with contrasting growth habits. Am-Eurasian J Agric Env Sci. 2010;9(5):495–9.

[35] Shaw AK, Bhardwaj PK, Ghosh S, Roy S, Saha S, Sherpa AR, et al. β-aminobutyric acid mediated drought stress alleviation in maize (Zea mays L.). Env Sci Pollut Res. 2016;23(3):2437–53. 10.1007/s11356-015-5445-z 26416125

[36] Singh T, Aspinall D, Paleg L. Proline accumulation and varietal adaptability to drought in barley: A potential metabolic measure of drought resistance. Nat N Biol. 1972;236(67):188–90. 10.1038/newbio236188a0 4503738

[37] Matile P, Hörtensteiner S, Thomas H. Chlorophyll degradation. Annu Rev Plant Physiol. 1999;50(1):67–95. 10.1146/annurev.arplant.50.1.67 15012204

[38] Kharrazi MAS, Rad MRN. Evaluation of sorghum genotypes under drought stress conditions using some stress tolerance indices. Afr J Biotechnol. 2011;10(61):13086–9.

[39] Okasha SA, Mubarak MH. Evaluation of some sugar beet genotypes under drought stress based on selection indices. J Agron Res. 2018;1(1):34–8.

[40] Golabadi M, Arzani A, Maibody SM. Assessment of drought tolerance in segregating populations in durum wheat. Afr J Agric Res. 2006;1(5):162–71.

[41] Rauf M, Munir M, ul Hassan M, Ahmad M, Afzal M. Performance of wheat genotypes under osmotic stress at germination and early seedling growth stage. Afr J Biotechnol. 2007;6(8).

[42] Link W, Hocking TJ, Stoddard FL. Evaluation of physiological traits for improving drought tolerance in faba bean (Vicia faba L.). Plant Soil. 2007;292(1):205–17.

[43] Khazaei H, Street K, Bari A, Mackay M, Stoddard FL. The FIGS (Focused Identification of Germplasm Strategy) approach identifies traits related to drought adaptation in Vicia faba genetic resources. PLoS One. 2013;8(5):e63107. 10.1371/journal.pone.0063107 PMC3648475 23667581

[44] Lawlor DW, Cornic G. Photosynthetic carbon assimilation and associated metabolism in relation to water deficits in higher plants. Plant Cell Env. 2002;25(2):275–94. 10.1046/j.0016-8025.2001.00814.x 11841670

[45] Sinclair T, Ludlow M. Who taught plants thermodynamics? The unfulfilled potential of plant water potential. Funct Plant Biol. 1985;12(3):213–7.

[46] Kaiser WM. Effects of water deficit on photosynthetic capacity. Physiol Plant. 1987;71(1):142–9.

[47] França MGC, Thi ATP, Pimentel C, Rossiello ROP, Zuily-Fodil Y, Laffray D. Differences in growth and water relations among Phaseolus vulgaris cultivars in response to induced drought stress. Env Exp Bot. 2000;43(3):227–37. 10.1016/s0098-8472(99)00060-x 10725522

[48] Rahbarian R, Khavari-Nejad R, Ganjeali A, Bagheri A, Najafi F. Drought stress effects on photosynthesis, chlorophyll fluorescence and water relations in tolerant and susceptible chickpea (Cicer arietinum L.) genotypes. Acta Biol Crac Ser Bot. 2011;53:47–56.

[49] Talebi R, Ensafi MH, Baghebani N, Karami E, Mohammadi K. Physiological responses of chickpea (Cicer arietinum) genotypes to drought stress. Environ. Exp Biol. 2013;11(1):9–15.

[50] Venekamp J, Lampe J, Koot J. Organic acids as sources for drought-induced proline synthesis in field bean plants, Vicia faba L. J Plant Physiol. 1989;133(6):654–9.

[51] Ammar M, Anwar F, El‐Harty E, Migdadi H, Abdel‐Khalik S, Al‐Faifi S, et al. Physiological and yield responses of faba bean (Vicia faba L.) to drought stress in managed and open field environments. J Agron Crop Sci. 2015;201(4):280–7.

[52] Rai V, Sharma U. Amino acids can modulate ABA induced stomatal closure, stomatal resistance and K+ fluxes in Vicia faba leaves. Beitr z Biol d Pflanz. 1991;66:393–405.

[53] Rajagopal V. The influence of exogenous proline on the stomatal resistance in Vicia faba. Physiol Plant. 1981;52(2):292–6.

[54] Hatmi S, Gruau C, Trotel-Aziz P, Villaume S, Rabenoelina F, Baillieul F, et al. Drought stress tolerance in grapevine involves activation of polyamine oxidation contributing to improved immune response and low susceptibility to Botrytis cinerea. J Exp Bot. 2015;66(3):775–87. 10.1093/jxb/eru436 25385768

